# Dates and Rates of Tick-Borne Encephalitis Virus—The Slowest Changing Tick-Borne Flavivirus

**DOI:** 10.3390/ijms24032921

**Published:** 2023-02-02

**Authors:** Artem N. Bondaryuk, Nina V. Kulakova, Olga I. Belykh, Yurij S. Bukin

**Affiliations:** 1 Laboratory of Natural Focal Viral Infections, Irkutsk Antiplague Research Institute of Siberia and the Far East, 664047 Irkutsk, Russia; 2 Limnological Institute, Siberian Branch of the Russian Academy of Sciences, 664033 Irkutsk, Russia; 3 Department of Biodiversity and Biological Resources, Siberian Institute of Plant Physiology and Biochemistry, Siberian Branch of the Russian Academy of Sciences, 664033 Irkutsk, Russia

**Keywords:** TBEV, tick-borne encephalitis virus, substitution rate, phylogenetics, population dynamics, divergence time, tMRCA

## Abstract

We evaluated the temporal signal and substitution rate of tick-borne encephalitis virus (TBEV) using 276 complete open reading frame (ORF) sequences with known collection dates. According to a permutation test, the TBEV Siberian subtype (TBEV-S) data set has no temporal structure and cannot be applied for substitution rate estimation without other TBEV subtypes. The substitution rate obtained suggests that the common clade of TBEV (TBEV-common), including all TBEV subtypes and louping-ill virus (LIV), is characterized by the lowest rate (1.87 × 10^−5^ substitutions per site per year (s/s/y) or 1 nucleotide substitution per ORF per 4.9 years; 95% highest posterior density (HPD) interval, 1.3–2.4 × 10^−5^ s/s/y) among all tick-borne flaviviruses previously assessed. Within TBEV-common, the TBEV European subtype (TBEV-E) has the lowest substitution rate (1.3 × 10^−5^ s/s/y or 1 nucleotide substitution per ORF per 7.5 years; 95% HPD, 1.0–1.8 × 10^−5^ s/s/y) as compared with TBEV Far-Eastern subtype (3.0 × 10^−5^ s/s/y or 1 nucleotide substitution per ORF per 3.2 years; 95% HPD, 1.6–4.5 × 10^−5^ s/s/y). TBEV-common representing the species *tick-borne encephalitis virus* diverged 9623 years ago (95% HPD interval, 6373–13,208 years). The TBEV Baikalian subtype is the youngest one (489 years; 95% HPD, 291–697 years) which differs significantly by age from TBEV-E (848 years; 95% HPD, 596–1112 years), LIV (2424 years; 95% HPD, 1572–3400 years), TBEV-FE (1936 years, 95% HPD, 1344–2598 years), and the joint clade of TBEV-S (2505 years, 95% HPD, 1700–3421 years) comprising Vasilchenko, Zausaev, and Baltic lineages.

## 1. Introduction

Tick-borne encephalitis virus (TBEV) is a pathogenic and neurotropic RNA-virus belonging to the family *Flaviviridae*, genus *Flavivirus* with up to 12,000 tick-borne encephalitis (TBE) cases identified annually from countries where the disease is tracked [1]. The coding region of the TBEV genome consists of one open reading frame (ORF) with a length of 10,245 nt encoding three structural and seven non-structural proteins [2] that is typical for flaviviruses [3].

The common clade of TBEV (TBEV-common) comprises at least five main subtypes: Far-Eastern (TBEV-FE), European (TBEV-E), Siberian (TBEV-S), Baikalian (TBEV-B) [4] and the recently discovered Himalayan (TBEV-H) [5] subtype. Moreover, the scientific community also distinguishes TBEV subtypes into subvariants or subgenotypes: within TBEV-S there is delineation into the Zausaev (TBEV-SZ), Vasilchenko (TBEV-SV), and Baltic (TBEV-SB) subvariants. Moreover, many distinct lineages with relatively long patristic distances are reported (e.g., Omskaya and Bosnia lineages [6] which are close to TBEV-S; the 178-79 strain, close to TBEV-B and -FE; the NL lineage, close to TBEV-E [7], etc.), but it is difficult to reach a consensus about their intraspecies status due to the lack of an accepted definition of subtype. Nevertheless, a 10% nucleotide difference threshold for TBEV subtypes was recently proposed [8]. In addition, louping-ill virus (LIV) primarily circulating in the British Isles is a paraphyletic unit in the structure of TBEV-common [9] and in the term of monophyly can be considered as a member of the *tick-borne encephalitis virus* species [10] or together with TBEV-E as a single species [11].

Several estimates of TBEV substitution rate based on the different loci (mainly, E gene or complete ORF sequences) have been reported with the values in different studies by an order of magnitude (e.g., median values of 0.8–1.4 × 10^−4^ for the E gene [12] and 3.9 × 10^−5^ for the complete ORF [9]). There was only one study, to our knowledge, where the temporal signal (or temporal structure [13,14,15]) of TBEV was evaluated [12] but the researchers analyzed E gene sequences (1488 nt) and used an informal root-to-tips regression analysis which tended to give false negative results [15] in the case of an ancient root and relatively narrow sampling windows (time between the most recent and oldest samples in a data set). Thus, it is possible that some of those estimates need to be revised.

In the current work, we aimed to assess the temporal structure of TBEV-common as well as TBEV subtypes separately using complete genome data available at present. We approached this by applying informal regression analysis in TempEst and a formal permutation test when collection dates between samples were permuted. Analysis suggests that TBEV-common representing the *tick-borne encephalitis virus* species is the oldest and the slowest virus among tick-borne flaviviruses. To the best of the authors’ knowledge, this work is the first comprehensive study of the TBEV substitution rate and population dynamics based on the actual complete ORF data set.

## 2. Results

### 2.1. Temporal Signal in the Data Sets of the Common Clade and Different Subtypes of TBEV

**Figure 1:**
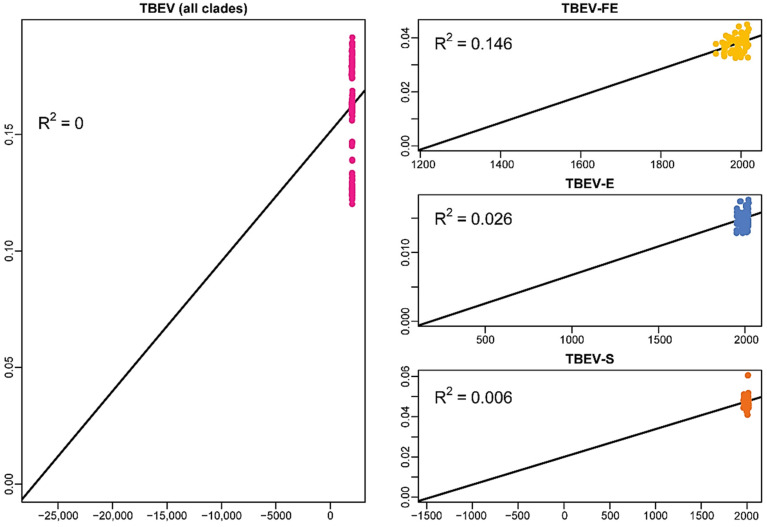
Root-to-tips regression analysis indicating a correlation between genetic distances from a root to tree tips and collection dates of samples. The X-axes represent calendar years where negative values are years BC. The abbreviates in the titles of windows are the common tick-borne encephalitis virus clade (TBEV-common) and TBEV subtypes: Far-Eastern (-FE), European (-E), and Siberian (-S).

We assessed the temporal signal using the complete genome data set of the common TBEV clade including LIV and the separate data sets of each TBEV subtype. LIV was not analyzed individually since it has been previously demonstrated with the permutation test that LIV has no temporal structure [16]. Preliminary analysis with TempEst showed (Figure 1) the lack of a correlation between the collection dates and root-to-tips genetic distances in all data sets except TBEV-FE with R^2^ = 0.145 (weak temporal structure).

**Figure 2:**
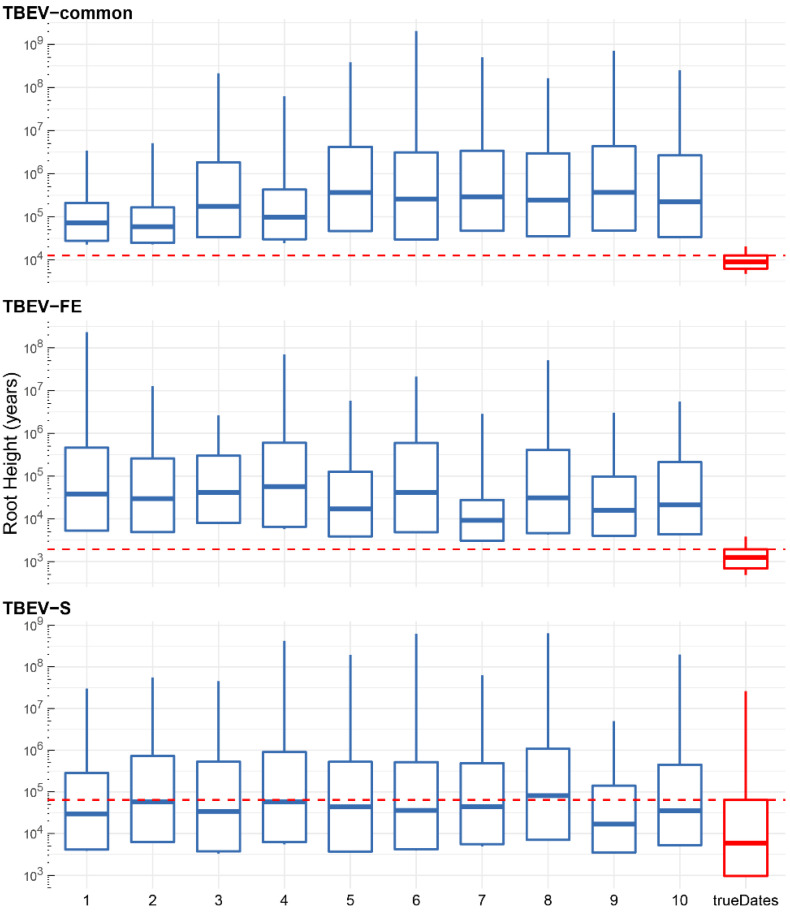
Permutation test results for the common clade of tick-borne encephalitis virus (TBEV-common), Far-Eastern (-FE) and Siberian (-S) subtypes of TBEV. Log Y-axes represent root height values in years before the most recent sample (2 June 2021). Blue boxplots indicate 10 pseudo-replicas with permuted dates in each analysis, and red boxplots represent MCMC runs with true collection dates for each sample/sequence. Red dashed lines show the upper limit of 95% highest posterior density (HPD) intervals of analyses with true dates.

As TempEst tends to infer false negative results (low R^2^ values) in the case of relatively old tree roots and narrow sampling windows [15], we relied on the permutation test with ten permuted replicas in each analysis. The permutation test (Figure 2) revealed that the data set comprising TBEV-common samples has a strong temporal signal without overlapping root height 95% HPD intervals of true dates analysis and permuted replicas, despite an extremely low R^2^ of 3 × 10^−5^ inferred by TempEst (see Figure 1).

Moreover, TBEV-FE but not TBEV-S demonstrated the presence of temporal structure. The results obtained validate the use of the common TBEV clade and TBEV-FE complete genome data sets in molecular clock analysis. Thus, TBEV-S is not considered for substitution rate evaluation. For the population dynamics analysis of TBEV-S, we used the substitution rate of TBEV-common. TBEV-E was not used for the permutation test as it was shown previously that the TBEV-E data set contained the temporal signal [16].

### 2.2. Nucleotide Saturation Test

The nucleotide saturation analysis showed that the codons in the TBEV-common data set were not critically saturated by substitutions (Iss = 0.271 and Iss.c = 0.571) and, therefore, were applicable for phylogenetic analysis.

### 2.3. Substitution Rates

**Figure 3:**
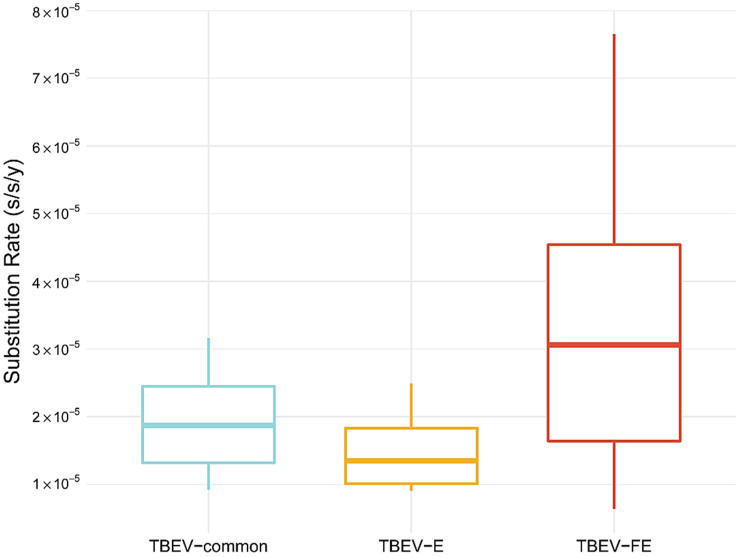
Comparing the substitution rates of the common clade of tick-borne encephalitis virus (TBEV-common), the European (TBEV-E) and Far-Eastern (TBEV-FE) subtypes. The Siberian subtype of TBEV (TBEV-S) was not included in the analysis since it had no temporal structure according to permutation test results.

Substitution rate estimates in BEAST indicated that TBEV-E was characterized by the lowest substitution rate (1.3 × 10^−5^ substitutions per site per year (s/s/y) or 1 nucleotide substitution per ORF per 7.5 years; 95% HPD, 1.0–1.8 × 10^−5^ s/s/y) in comparison with TBEV-common (1.87 × 10^−5^ s/s/y or 1 nucleotide substitution per ORF per 4.9 years; 95% HPD, 1.3–2.4 × 10^−5^ s/s/y) and TBEV-FE (3.0 × 10^−5^ s/s/y or 1 nucleotide substitution per ORF per 3.2 years; 95% HPD, 1.6–4.5 × 10^−5^ s/s/y), with a slight overlap of 95% HPD intervals (Figure 3). TBEV-FE has the highest substitution rate but the widest 95% HPD interval.

The UCLD clock analysis exhibited 95% of TBEV-E CV posterior density being lower than 0.13, which implied that the data is clock-like. TBEV-common and TBEV-FE demonstrated non-clock-like behavior with CV ranging 0.27–0.42 and 0.34–0.57, respectively, which supports applying UCLD.

### 2.4. Divergence Times

**Figure 4:**
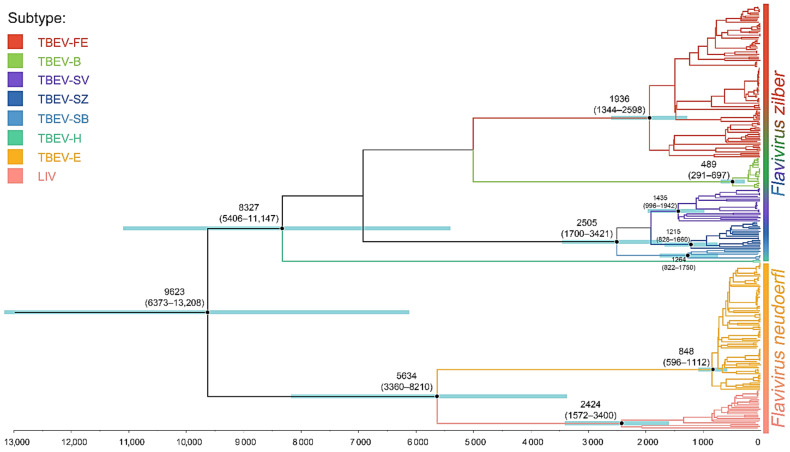
Maximum clade credibility tree of tick-borne encephalitis virus (TBEV) aggregated by FigTree from trees produced by an MCMC run. Horizontal bars are 95% highest posterior probability density (HPD) intervals. Numbers above main nodes indicate divergence times before the most recent sample collection (2 June 2021) with 95% HPD in parenthesis. All nodes dated on the tree have a posterior probability of 1.0.

To evaluate the divergence time of TBEV subtypes, we used the data set containing 276 complete genome sequences of TBEV-common. The results of our analysis in BEAST suggest that the root age of TBEV-common is 9623 years (95% HPD, 6373–13,208 years) (Figure 4).

**Figure 5:**
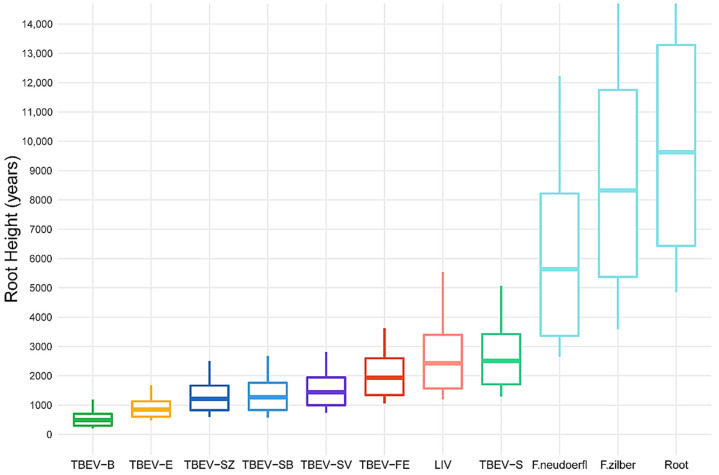
Boxplots of tick-borne encephalitis virus (TBEV) subtype ages in years before the most recent sample (2 June 2021). The X-axis is labeled by subtypes of TBEV: Baikalian (-B), European (-E), Siberian (-S) with Zausaev (-SZ), Baltic (-SB), and Vasilchenko (-SV) subgenotypes, Far-Eastern (-FE), also with louping-ill virus (LIV), *Flavivirus neudoerfl,* and *Flavivrus zilber* clades. The root is the common clade of TBEV (TBEV-common). The edges of boxplots indicate 95% highest posterior density (HPD) intervals. The upper limit of *F. neudoerfl*, *F. zilber*, and root ages are restricted for convenience.

The youngest TBEV subtype is TBEV-B (489 years; 95% HPD, 291–697 years); however, these estimates should be treated with caution, as the number of TBEV-B complete genome sequences (20) is relatively small (Figure 5).

Median values are listed in ascending order as follows: TBEV-E (848 years; 95% HPD, 596–1112 years); the subvariants of TBEV-S: TBEV-SZ (1215 years; 95% HPD, 828–1660 years), TBEV-SB (1264 years; 95% HPD, 822–1750 years), TBEV-SV (1435 years; 95% HPD, 996–1942 years); TBEV-FE (1936 years, 95% HPD, 1344–2598 years); LIV (2424 years; 95% HPD, 1572–3400 years); the joint clade of TBEV-S (2505 years, 95% HPD, 1700–3421 years); the monophyletic species *Flavivirus neudoerfl* (5634 years; 95% HPD, 3360–8210 years) and *Flavivirus zilber* (8327 years; 95% HPD, 5406–11,147 years); the tree root (9623 years; 95% HPD, 6373–13,208 years).

The ages of the following TBEV subtypes (in ascending order) are significantly different: TBEV-B, -E, LIV and -FE/-S (95% HPD intervals of LIV, TBEV-FE and -S are overlapped substantially).

### 2.5. Relative Genetic Diversity

**Figure 6:**
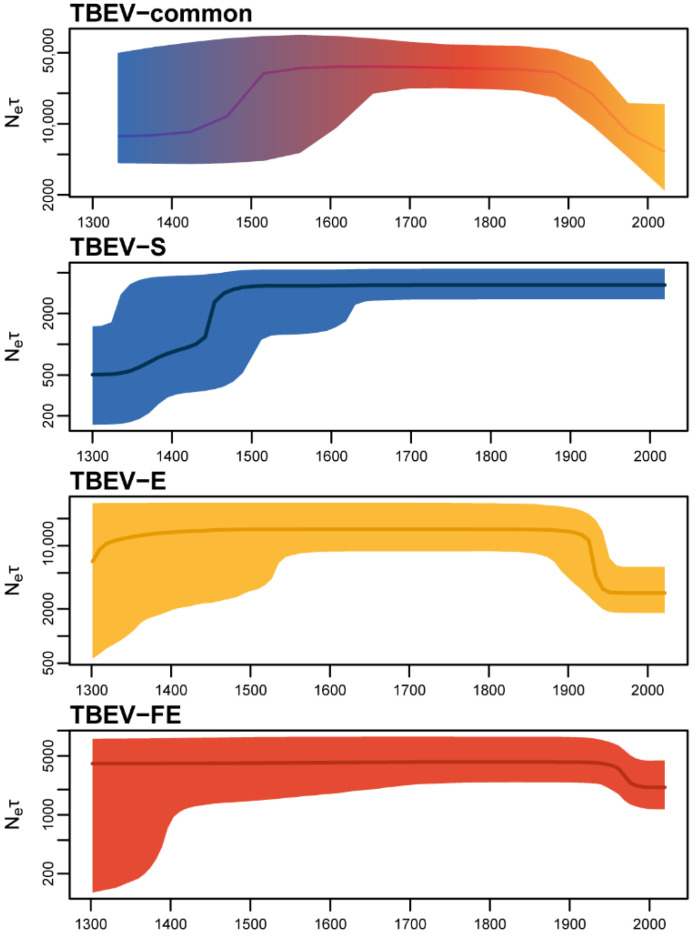
Population dynamics of the common clade of tick-borne encephalitis virus (TBEV-common), TBEV Siberian subtype (TBEV-S), European subtype (TBEV-E), and Far-Eastern subtype (TBEV-FE). The log Y-axis indicates relative genetic diversity—N_e_ × τ, where N_e_ is an effective population size or in the context of infectious diseases it is the effective number of infections [17], τ is the generation time or time between successive infections in transmission chains. The solid lines are median values, filled areas are 95% highest posterior density (HPD) intervals. To reconstruct the population dynamics of TBEV-S, we applied substitution rate estimates obtained during the analysis of TBEV-common. The gradient fill of TBEV-common displays features in the N_e_ × τ trend shared with the other TBEV subtypes.

Bayesian skyline reconstruction of TBEV-common with the strongest population structure (i.e., incorporating samples of main TBEV subtypes such as TBEV-S, -E, -FE, -B, that are isolated geographically) revealed population dynamics with patterns similar to the skyline trends of TBEV-S, -E, and -FE, separately (Figure 6).

In other words, the N_e_ × τ trend of TBEV-common consists of the TBEV-S N_e_ × τ uprising in the 1300s followed by the TBEV-S, -E, -FE stable period during the years 1500–1900, and the decline of TBEV-E and -FE relative genetic diversity values in the 1900s. Notably, the TBEV-S skyline trend has no decline close to the present.

## 3. Discussion

In the previous studies, analyses of a relatively small set of TBEV ORF nucleotide sequences (from 36 to 39 operation taxonomic units (OTUs)) inferred similar root ages: 3119 years (95% HPD, 1123–7695 years) [5], 3369 years (95% HPD, 3183–6318 years) [9] and 3118 years (95% HPD, 1775–4877 years) [18]. Those roots are three times younger than we reported here. In our previous work [19], scrutinizing 164 TBEV complete genome sequences revealed the older TBEV root to be 9900 years (95% HPD, 6400–14,800 years), which is comparable with estimates derived in the current study with 276 OTUs, i.e., 9280 years (95% HPD, 6190–12,800 years). We hypothesized that the data set of at least 164 TBEV complete genome sequences is sufficient to infer stable root age values.

Unfortunately, only one study mentioned above (34 TBEV complete genome sequences) reported on the substitution rate of 4 × 10^–5^ s/s/y (95% HPD, 2.6–5.3 × 10^–5^ s/s/y) [9], which is significantly different from the analysis of 164 OTUs (95% HPD, 1.0–2.2 × 10^–5^ s/s/y) [19] and the results of the current study (276 OTUs) (1.9 × 10^–5^ s/s/y; 95% HPD, 1.3–2.5 × 10^–5^ s/s/y). Interestingly, Uzcátegui, et al. (2012) [9] obtained the substitution rate for the TBEV-S + TBEV-FE clade (so-called *Ixodes persulcatus*-carrying viruses) with the very wide 95% HPD interval, 3.63 × 10^−9^–3.63 × 10^−5^ s/s/y, that is quite similar to our evaluation of the TBEV-S substitution rate without a temporal signal (95% HPD interval, 1.7 × 10^−9^–1.9 × 10^−5^ s/s/y). It is likely that the data set analyzed in the study of Uzcátegui, et al. (2012) [9] (the clade of *I. persulcatus*-carrying viruses, specifically) also has no temporal structure since the number of samples of the TBEV-FE + TBEV-S (24) clade is smaller than we used in this work for TBEV-S (45 OTUs).

**Figure 7:**
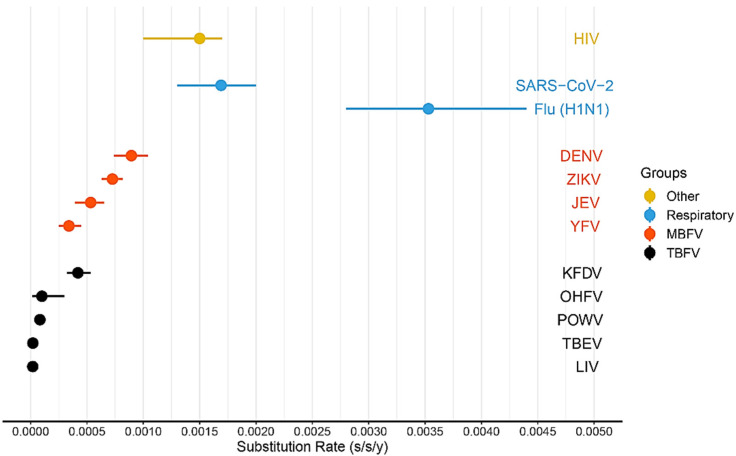
Nucleotide substitution rate comparison of tick-borne flaviviruses (TBFV), mosquito-borne flaviviruses (MBFV), respiratory viruses and HIV. Abbreviations: LIV—louping-ill virus, TBEV—tick-borne encephalitis virus, POWV—Powassan virus, OHFV—Omsk hemorrhagic fever virus, KFDV—Kyasanur Forest disease virus, YFV—yellow fever virus, JEV—Japanese encephalitis virus, ZIKV—Zika virus, DENV—dengue virus. The filled circles are median values, the horizontal bars are 95% highest posterior density (HPD) intervals.

The TBEV substitution rate calculated in this study (95% HPD, 1.4–2.6 × 10^–5^ s/s/y) is the lowest as compared with the other tick-borne flavivirus species (Powassan virus: 95% HPD, 8.2–10.4 × 10^−5^ s/s/y [20]; Omsk hemorrhagic fever virus: 1.3 × 10^−5^ –3.0 × 10^−4^ with mean 1.3 × 10^−4^ (too wide a HPD interval, a data set does not seem to have temporal structure) [21]; Kyasanur Forest disease virus, 3.2–5.3 × 10^−4^ [22]) (Figure 3). In general, tick-borne flaviviruses evolve slower than mosquito-borne flaviviruses (Figure 7), such as Zika virus (95% HPD, 6.2–8.2 × 10^−4^ s/s/y [23]), dengue virus (95% HPD, 7.4 × 10^−4^–1.0 × 10^−3^ [24]), Japanese encephalitis virus (95% HPD 3.9–6.5 × 10^−4^ [25]), yellow fever virus (95% HPD, 2.5–4.5 × 10^−4^ [26]), respiratory viruses (SARS-CoV-2: 95% HPD, 1.3–2.0 × 10^−3^ [27]; influenza virus H1N1: 95% HPD, 2.8–4.4 × 10^–3^ [28]), and HIV (95% HPD, 1.0–1.7 × 10^−3^ [29]).

Some researchers hypothesized that such a low substitution rate is largely due to the constraining effect imposed by the two-host system (invertebrates and vertebrates), extended tick lifecycle (3–5 years) relative to mosquitoes, and the relatively low virus titers with a much more rapid lifecycle in mosquitoes [30]. Our estimates are also much lower than those obtained by the analysis of E gene sequences (mean values for different subtypes ranged from 0.89 × 10^−4^ to 1.44 × 10^−4^) [12].

Our data showed that TBEV was the oldest tick-borne flavivirus diverged 9623 years ago (95% HPD, 6373–13,208) in comparison with Powassan virus (4170 years; 95% HPD, 2600–6030 years) [31]; Omsk hemorrhagic fever virus (704 years; 95% HPD, 65–1833 years) [21]; and Kyasanur Forest disease virus (704 years; 95% HPD, 65–1833 years, but the temporal signal was not evaluated) [22] (Figure 5).

Previously [11], we proposed to unite the clades of TBEV-FE, -B, -S, -H, and other single TBEV lineages into the separate virus species *F. zilber*, and the TBEV-E subtype with LIV into another species *F. neudoerfl*. This is required by the monophyletic conception (Figure 4) and the ICTV definition of virus species (https://ictv.global/about/code; accessed on 26 December 2022). Our estimates suggest *F. neudoerfl* is slightly younger than *F. zilber* with the time to the most recent common ancestor being 5634 and 8327 years, respectively; however, the 95% HPD intervals are significantly overlapped.

TBEV-common with a strong population structure has demonstrated population dynamics features shared with three main subtypes (TBEV-S, -E, -FE; Figure 6), which highlights insidious aspects of coalescent inference assumptions (such as population structure or isolation influence). In the case of TBEV-common, Bayesian Skyline does not reflect the true changes of relative genetic diversity; therefore, during Bayesian Skyline analysis, TBEV-common as a metapopulation should be subdivided into subpopulations (or demes) according to TBEV subtypes. When analyzing TBEV subtypes separately, it was revealed that only the TBEV-S population has pronounced N_e_ × τ ascending in the early 1300s and has no N_e_ × τ decline to the present. In a previous study [12], researchers used the Bayesian SkyGrid model to analyze 551 TBEV E gene nucleotide sequences and obtained an opposite pattern of population dynamics of three main TBEV subtypes (TBEV-S, -E, -FE) with ascending relative genetic diversity up to the present. This discrepancy is due to the difference in molecular markers compared to parameters of the Skyline and SkyGrid models. We believe that an increase in the number of loci (TBEV ORF contains ten genes) has a better effect on the accuracy of the population dynamics inference than an increase in the number of sequences. This is at least known for the Extended Bayesian Skyline Plot [32]. Evaluation of the number of parsimony-informative sites in the E gene data set with known collection dates (723 samples) and the ORF data set (276 samples) used in the current study showed that despite a relatively low number of sequences, the ORF data set contained almost six times more informative sites than E gene alone (4082 and 658 or 14.8 and 0.9 informative sites per OTU, respectively).

The observed dynamics of relative genetic diversity is associated with changes in the two-host vertebrate–invertebrate (rodent-tick) system. In turn, the climatic changes during this period could have affected rodents and ticks. The time period from the 1300s to the end of the 1900s is known as the Little Ice Age [33,34]. From 950 to 1250, there was a medieval climatic optimum with, respectively, high temperature and humidity in the northern hemisphere [34] followed by a sharp cooling at the first stage of the Little Ice Age (1300–1440) in the Eurasian part of the TBEV distribution area. All three TBE subtypes had, respectively, high relative genetic diversity during this first stage. At the second stage of the Little Ice Age (1440–1560), there was a slight rise in temperature. The third stage (1560–1900) was characterized by a drop in the temperature and humidity associated with the period of low solar activity [34,35]. The Little Ice Age ended at the beginning of the 1900s and since then there has been a rise in temperature in the northern hemisphere. The TBEV-S population increase was detected in the first stage of the Little Ice Age. Some studies based on the historical observations [36] and the population genetic analysis concluded that there was a surge in the number of rodents during the Little Ice Age [37,38]. This surge, in turn, was associated with the liberation of ecological niches by larger mammals and a decrease in the number of predators including poikilotherms. A rise in the rodent population supports the increase of relative genetic diversity of TBEV. An increase in the relative genetic diversity of TBEV-S during the Little Ice Age could also be due to the retention of the sufficient level of humidity on the Eurasian continent [39,40,41]. The consequence of this was the preservation of the suitable habitats for ticks. The decline in the effective population sizes of TBEV-E and TBEV-FE after 1900 is probably associated with the following:

- Hidden population structure within TBEV-E and -FE (so-called confounding effect of population structure [42]);
- Large-scale forest treatment by acaricides including dichlorodiphenyltrichloroethane (DDT) to prevent human infection.

The TBEV-S effective population size shows no decline up to the present due to either a relatively small sample size of TBEV-S or a weaker population structure.

## 4. Materials and Methods

### 4.1. Data Set Design

We searched homologous ORF nucleotide sequences of TBEV with known collection dates in the GenBank database. LIV was also included in the search since together with TBEV it formed a paraphyletic clade in the phylogenetic tree. In total, 276 ORF sequences (10,242 nt) were found and filtered by stop codons. The data set composition is presented in Table 1.

Single TBEV lineages (e.g., 178-79 strain, Bosnia and Omskaya lineages of TBEV-S, NL lineage of TBEV-E) were not combined with the main TBEV subtypes in a union clade due to relatively high patristic distances, and, therefore, were not involved in the evaluation of divergence times.

### 4.2. Codon Saturation Measuring

The index of substitution saturation (I_ss_) in a set of aligned nucleotide sequences was calculated in DAMBE [44] and compared with critical I_ss_ values (referred to as I_ss.c_ [45]). Minor saturation was observed in the case of I_ss_ < I_ss.c_.

### 4.3. Temporal Signal Assessment

We assessed temporal signal in the TBEV-common data set using all 276 ORF sequences along with subtypes of TBEV separately. TBEV-S comprised TBEV-SB, -SV, and -SZ variants. We did not evaluate the signal of TBEV-E since it was previously approved with a permutation test by Clark, et al. (2020) [16]. The evidence of a lack of the temporal structure in the LIV complete genome data set was also shown by these authors. Thus, temporal structure was evaluated for TBEV-common, TBEV-FE and TBEV-S data sets.

Firstly, we used TempEst [46] to conduct regression analysis where the correlation between the genetic distances from a tree root to tips (root-to-tips distances) and the collection dates was estimated. This method is informal and tends to give false negative results in the case when a sampling window (Table 1) is narrow relative to root height [15]. Secondly, we then applied intuitive and formal permutation tests where collection dates were permuted between samples. We used 10 permuted replicas, which were analyzed in BEAST. Root height values obtained were compared with the analysis of data sets with true collection dates. In the case of overlapping 95% HPD intervals of true and permuted dates, temporal signal was taken as negative and vice versa. To reduce the computational cost, MCMC runs of permuted replicas were continued until effective sample sizes (ESS) of all parameters reached 100.

### 4.4. Evolution Model Selection

To choose the substitution model, we used ModelFinder [47] implemented in IQTREE v.1.6.12 [48].

A molecular clock model was tested by the coefficient of rate variation (CV) values according to an empirical rule [49] when CV posterior density in the range 0.0–0.1 indicates clock-likeness or strict clock (SC) evidence and, otherwise, a relaxed clock with uncorrelated lognormal prior distributions (UCLD) of substitution rates among lineages. Substitution rate values are indicated in substitutions per site per year (s/s/y) with median values and 95% HPD intervals.

### 4.5. Phylogenetic Analysis in BEAST

Phylogenetic analyses were performed in BEAST v.2.6.3 [50]. Markov chain Monte Carlo (MCMC) analyses were run until ESS values of all parameters were >200 with a sampling frequency at which the total number of trees was at least 40,000. The longest MCMC length of more than one billion iterations was for the TBEV-common data set. MCMC convergence was checked in Tracer v.1.7.2 [51]. Most credibility clade (MCC) trees were estimated with TreeAnnotator.

The BEAST files and fasta alignment used in this study can be found at link: https://doi.org/10.6084/m9.figshare.21780239 (accessed on 26 December 2022)

### 4.6. Bayesian Skyline Reconstruction

To reconstruct population dynamics, we used the Bayesian Skyline method [52] with default priors. Due to the strong population structure of TBEV-common, we additionally subdivided the TBEV-common data set into TBEV-S, -E, and -FE subtypes to compare population dynamics inferences of populations with weak and strong population structures. As TBEV-S showed the lack of temporal signal in the permutation test, in TBEV-S population dynamics analysis, we applied the substitution rate of TBEV-common.

## 5. Conclusions

1. TBEV has the lowest substitution rate among other tick-borne flavivirus species;
2. The actual data set of TBEV-S ORF sequences has no temporal structure;
3. TBEV common clade is characterized by the lowest substitution rate (1.87 × 10^−5^ s/s/y or 1 nucleotide substitution per ORF per 4.9 years; 95% HPD, 1.3–2.4 × 10^−5^ s/s/y) in comparison with the other tick-borne flaviviruses;
4. TBEV-E has a lower evolutionary rate (1.3 × 10^−5^ s/s/y or 1 nucleotide substitution per ORF per 7.5 years; 95% HPD, 1.0–1.8 × 10^−5^ s/s/y) compared with TBEV-common (1.87 × 10^−5^ s/s/y or 1 nucleotide substitution per ORF per 4.9 years; 95% HPD, 1.3–2.4 × 10^−5^ s/s/y) and TBEV-FE (3.0 × 10^−5^ s/s/y or 1 nucleotide substitution per ORF per 3.2 years; 95% HPD, 1.6–4.5 × 10^−5^ s/s/y);
5. TBEV-common representing the species *tick-borne encephalitis virus* according to median values diverged 9623 years ago with a 95% HPD interval of 6373–13,208 years;
6. TBEV-B is the youngest TBEV subtype occurred about 489 years ago (95% HPD, 291–697 years) that is significantly different from TBEV-E (848 years; 95% HPD, 596–1112 years), LIV (2424 years; 95% HPD, 1572–3400 years), TBEV-FE (1936 years, 95% HPD, 1344–2598 years), and the joint clade of TBEV-S (2505 years, 95% HPD, 1700–3421 years);
7. The population dynamics of TBEV-common as a metapopulation with a strong population structure has a “chimeric” pattern comprising features of dynamics of highly isolated TBEV subtypes (TBEV-S, -E, and -FE) so N_e_ × τ dynamics inferred from TBEV-common cannot be interpreted as changes in the effective number of infections.

## Figures and Tables

**Table 1:** The data set composition and model combination parameters.

Virus/Subtype ^1^	Number of Sequences	Sampling Window ^2^	Substitution Model ^3^	Molecular Clock ^4^
LIV	25	84	–	–
TBEV-B	20	26	–	–
TBEV-E	83	70	SRD06 [43]	SC
TBEV-FE	99	81	GTR + G + I	UCLD
TBEV-H	2	0	–	–
TBEV-SB	7	46	GTR + G + I	UCLD
TBEV-SV	22	58		
TBEV-SZ	18	58		
TBEV-common	276	90	GTR + G + I	UCLD

^1^ Abbreviations: LIV—louping-ill virus; TBEV-B—Baikalian subtype of tick-borne encephalitis virus, -FE—Far-Eastern subtype, -H—Himalayan subtype, -SB—Baltic lineage of Siberian subtype, -SV Vasilchenko lineage of Siberian subtype, -SZ—Zausaev lineage of Siberian subtype, TBEV-common—the common clade of TBEV; ^2^ Sampling window is a difference in years between the most recent and oldest collection dates of samples; ^3^ Substitution model was chosen according to BIC scores calculated with ModelFinder implemented in IQTREE except for TBEV which was analyzed previously [43] by the SRD06 model. If a data set is not analyzed separately, a cell contains “–”. ^4^ The molecular clock model was chosen according to the coefficient of variation (CV) values calculated with preliminary BEAST runs with an uncorrelated relaxed lognormal (UCLD) clock.

## Data Availability

The BEAST files and fasta alignment used in this study can be found at link: https://doi.org/10.6084/m9.figshare.21780239 (accessed on 26 December 2022).
